# Blocking CD47 efficiently potentiated therapeutic effects of anti-angiogenic therapy in non-small cell lung cancer

**DOI:** 10.1186/s40425-019-0812-9

**Published:** 2019-12-11

**Authors:** Xuyao Zhang, Yichen Wang, Jiajun Fan, Wei Chen, Jingyun Luan, Xiaobin Mei, Shaofei Wang, Yubin Li, Li Ye, Song Li, Wenzhi Tian, Kai Yin, Dianwen Ju

**Affiliations:** 1 Minhang Hospital, Fudan University, 170 Xinsong Road, Shanghai, 201199 China; 2 Department of Microbiological and Biochemical Pharmacy, School of Pharmacy, Fudan University, Shanghai, 201203 China; 3 Department of Systems Pharmacology and Translational Therapeutics, Perelman School of Medicine, University of Pennsylvania, Philadelphia, 19104 USA; 4 Changhai Hospital, Second Military Medical University, Shanghai, 200433 China; 5 ImmuneOnco Biopharma (Shanghai) Co., Ltd., 1043 Halei Road, Shanghai, 201203 China

**Keywords:** Anti-angiogenesis, VEGF, CD47, Immunotherapy, Bispecific therapy

## Abstract

**Background:**

Inhibitors targeting VEGF and VEGFR are commonly used in the clinic, but only a subset of patients could benefit from these inhibitors and the efficacy was limited by multiple relapse mechanisms. In this work, we aimed to investigate the role of innate immune response in anti-angiogenic therapy and explore efficient therapeutic strategies to enhance efficacy of anti-angiogenic therapy against non-small cell lung cancer (NSCLC).

**Methods:**

Three NSCLC tumor models with responses to VEGF inhibitors were designed to determine innate immune-related underpinnings of resistance to anti-angiogenic therapy. Immunofluorescence staining, fluorescence-activated cell sorting and immunoblot analysis were employed to reveal the expression of immune checkpoint regulator CD47 in refractory NSCLC. Metastatic xenograft models and VEGFR1-SIRPα fusion protein were applied to evaluate the therapeutic effect of simultaneous disruption of angiogenetic axis and CD47-SIRPα axis.

**Results:**

Up-regulation of an innate immunosuppressive pathway, CD47, the ligand of the negative immune checkpoint regulator SIRPα (signal regulatory protein alpha), was observed in NSCLC tumors during anti-angiogenic therapy. Further studies revealed that CD47 upregulation in refractory lung tumor models was mediated by TNF-α/NF-κB1 signal pathway. Targeting CD47 could trigger macrophage-mediated elimination of the relapsed NSCLC cells, eliciting synergistic anti-tumor effect. Moreover, simultaneously targeting VEGF and CD47 by VEGFR1-SIRPα fusion protein induced macrophages infiltration and sensitized NSCLC to angiogenesis inhibitors and CD47 blockade.

**Conclusions:**

Our research provided evidence that CD47 blockade could sensitize NSCLC to anti-angiogenic therapy and potentiate its anti-tumor effects by enhancing macrophage infiltration and tumor cell destruction, providing novel therapeutics for NSCLC by disrupting CD47/SIRPα interaction and angiogenetic axis.

## Background

Sustained angiogenesis is an important hallmark of non-small cell lung cancer (NSCLC) [1]. A series of molecules has been identified to play crucial roles in angiogenesis and vasculogenesis, and most studies to date were focused on VEGFR (vascular endothelial growth factor receptor) and its ligand VEGF [2, 3]. The biological functions of VEGF and VEGFR in tumor angiogenesis provided a convincing principle for the development of inhibiting agents targeting VEGF-VEGFR axis [4]. Since the last decades, more than ten anti-angiogenic therapeutics including bevacizumab, regorafenib and sorafenib have been approved for the therapy against several malignant diseases [3–5]. Unfortunately, due to the unknown relapse mechanisms, beneficial effects of these drugs used as monotherapy or in combination with chemotherapy are only observed in limited number of patients [6–8]. Here, in this context, we aimed to elucidate novel relapse mechanisms underlying the anti-angiogenic therapy and provide efficient strategy to enhance the anti-tumor effect of anti-angiogenic treatment.

Studies of tumor immune microenvironment reveal that tumors evade immune system detection through developing the local angiogenic vasculature [9–12]. Angiogenic vasculature in tumors thwarts the extravasation of tumor-responsive lymphocytes and develops an immunosuppressive microenvironment that confers tumors to evade host’s immune surveillance [10, 13]. Increased VEGF in tumors impairs lymphocyte-endothelial interaction by decreasing intercellular cell adhesion molecules in neovascularization to block immune cells infiltration into the tumors [10]. Besides, VEGF can directly trigger regulatory T cells proliferation and inhibit the maturation of dendritic cells [14]. Stimulation of the host’s immune system with immune checkpoint inhibitors showed robust anti-tumor effects and hold promise for the treatment of malignant tumors [15, 16]. Considering the facts that tumor immune microenvironment is bound up with tumor angiogenic vasculature, effort has been made to investigate the relationship between anti-angiogenic therapy and tumor immunotherapy [17–19]. It was reported that anti-tumor effect of VEGF/VEGFR inhibitors was dependent on their abilities to elicit an immune-activated milieu in breast and pancreatic tumors. Combinational use of anti-PD-L1 treatment sensitized tumors to VEGF/VEGFR blockade and prolonged anti-tumor effect [9]. However, the important role of innate immune response, especially macrophage, in anti-angiogenic therapy was still unknown.

CD47 (Cluster of differentiation 47)/SIRPα (signal-regulatory protein alpha), an innate negative immune regulatory axis that transmits “don’t eat me” signal to macrophages and confers tumor cells resistant to immune surveillance [20–23]. CD47/SIRPα-based therapies have been proven as an effective treatment for solid tumors and hematologic malignancies, with several clinical trials including CD47-blocking monoclonal antibodies or SIRPα-Fc fusion protein [24–26]. These findings highlighted the great impetus in tumor immunotherapy to mobilize macrophages to participate in anti-tumor activities. Compared with other isomers of the VEGFR family, VEGFR1 showed very high binding affinity to VEGF and functioned as a decoy receptor to VEGF [4]. Aflibercept, a soluble chimeric protein based on the extracellular domain of VEGFR1, has been approved for the therapy of colorectal cancer [2]. In this context, we found for the first time that the unsustainable efficacy of anti-angiogenic therapy was resulted from their ability to upregulate CD47 expression in tumor microenvironment conferring NSCLC resistant to anti-angiogenic therapy. Administration of VEGF-VEGFR inhibitor VEGFR1-Fc in combination with CD47 blocking fusion protein generated synergistic antitumor efficacy, highlighting the potential therapeutic strategies for NSCLC via blocking angiogenetic axis and CD47/SIRPα anti-phagocytic axis.

## Methods

### Reagents

Reagents and antibodies were obtained as follows: anti-CA9 antibody (Novus Biologicals, Littleton, USA), carboxyfluorescein diacetate succinimidyl ester (CFDA SE) (Beyotime Biotech, Hangzhou, China), FITC-labeled anti-NF-κB1, PE-labeled anti-CD47, PerCP/Cyanine5.5-labeled anti-CD31, Alexa Fluor 488-labeled anti-CD11b, PE-labeled F4/80 and APC-labeled anti-CD45 antibodies (Biolegend, San Diego, USA), Bevacizumab (Roche Genentech, South San Francisco, USA). BAY 11–7082 (Selleckchem, Shanghai, China). Clodronate liposomes (FormuMax Scientific, Inc., Sunnyvale, USA). SIRPα-Fc fusion protein was expressed as previously described [27]. VEGFR1-SIRPα is based on the first extracellular domain of SIRPα and the second extracellular domain of VEGFR1. SIRPα-VEGFR1 expression cassette sequence was synthesized (GenBank accession number: MG920788), expressed and purified from CHO cells.

### Cell lines and culture conditions

NCI-H1975, A549 and LLC (Lewis Lung Carcinoma) cells were purchased from Cell Bank of Shanghai Institutes for Biological Sciences, Chinese Academy of Sciences and authenticated by short tandem repeat fingerprinting in the cell bank. Cells were cultured in medium with 10% FBS (Gibco, San Diego, USA) and passaged less than 6 months upon receipt.

### Fluorescence-activated cell sorting

VEGFR1-Fc was intraperitoneally injected into tumor-bearing mice twice a week for 4 weeks. Tumors were then harvested and processed into a single-cell suspension. Cells were treated with Fc-blocking antibody, stained with PerCP/Cyanine5.5-labeled anti-CD31, APC-labeled anti-CD45 and PE/Cyanine 7-labeled anti-keratin antibodies. Endothelial cells were sorted as CD45^−^CD31^+^keratin^−^ cells. Immune cells were identified as CD45^+^CD31^−^keratin^−^ cells and tumor cells were sorted as CD45^−^CD31^−^keratin^+^ cells. To isolate NF-κB1^+^ cells from tumors and sort them into endothelial cells, immune cells and tumor cells, cells were collected and stained with PerCP/Cyanine5.5-labeled anti-CD31 and APC-labeled anti-CD45 antibodies. The cells were than fixed with 4% paraformaldehyde and permeabilized with triton X-100, and then stained with FITC-labeled anti-NF-κB1antibody. Analyses of sorted cells from tumor-bearing mice were performed using at least three independent mice for each treatment condition.

### Phagocytosis and cytotoxicity assay

Macrophage phagocytosis and cytotoxicity were detected as described previously [26, 28]. Briefly, primary mouse macrophages were obtained from femurs of BALB/c nude mice and cultured in medium containing macrophage colony-stimulating factor (100 ng/ml) and FBS (10%). One week later, macrophages were collected and co-cultured with CFDA SE-labeled NSCLC cells. After SIRPα-Fc treatment, confocal microscopy was used to calculate the phagocytic index. Cytotoxicity was examined by CytoTox 96® Non-Radio. Cytotoxicity Assay (Promega, Madison, USA) at different effector: target cell ratio.

### Immunoblot analysis

After VEGFR1-Fc treatment, NSCLC tumor tissues were harvested and homogenized with RIPA lysis buffer. Equivalent amounts of the extracted protein were analyzed by SDS-PAGE gel electrophoresis. ImageJ Software was used to quantify densitometric values of resulting bands.

### Tumor models

To construct subcutaneous xenograft models, BALB/c nude mice (6 weeks old) were subcutaneously inoculated with NSCLC cells (5 × 10^6^). To establish metastatic xenograft models, Nude mice were injected with NSCLC cells (1 × 10^6^) via the tail vein. To construct syngeneic immunocompetent model, C57BL/6 mice were subcutaneously inoculated with 1 × 10^6^ of LLC cells. VEGFR1-Fc (10 mg/kg), SIRPα-Fc (10 mg/kg) and VEGFR1-SIRPα (10 mg/kg) were injected intraperitoneally twice a week. BAY 11–7082 (5 mg/kg) was injected intraperitoneally three times a week. Clo/liposome (200 μl per mouse) was injected intraperitoneally twice a week.

### Statistical analysis

GraphPad Prism 7 was employed to analyze the data. Comparison in this study was performed by Student’s t-test or One-Way ANOVA analysis. *P* value < 0.05 was regarded as statistical significance.

## Results

### CD47 expression increased in NSCLC relapsing from anti-angiogenic treatment

**Fig. 1:**
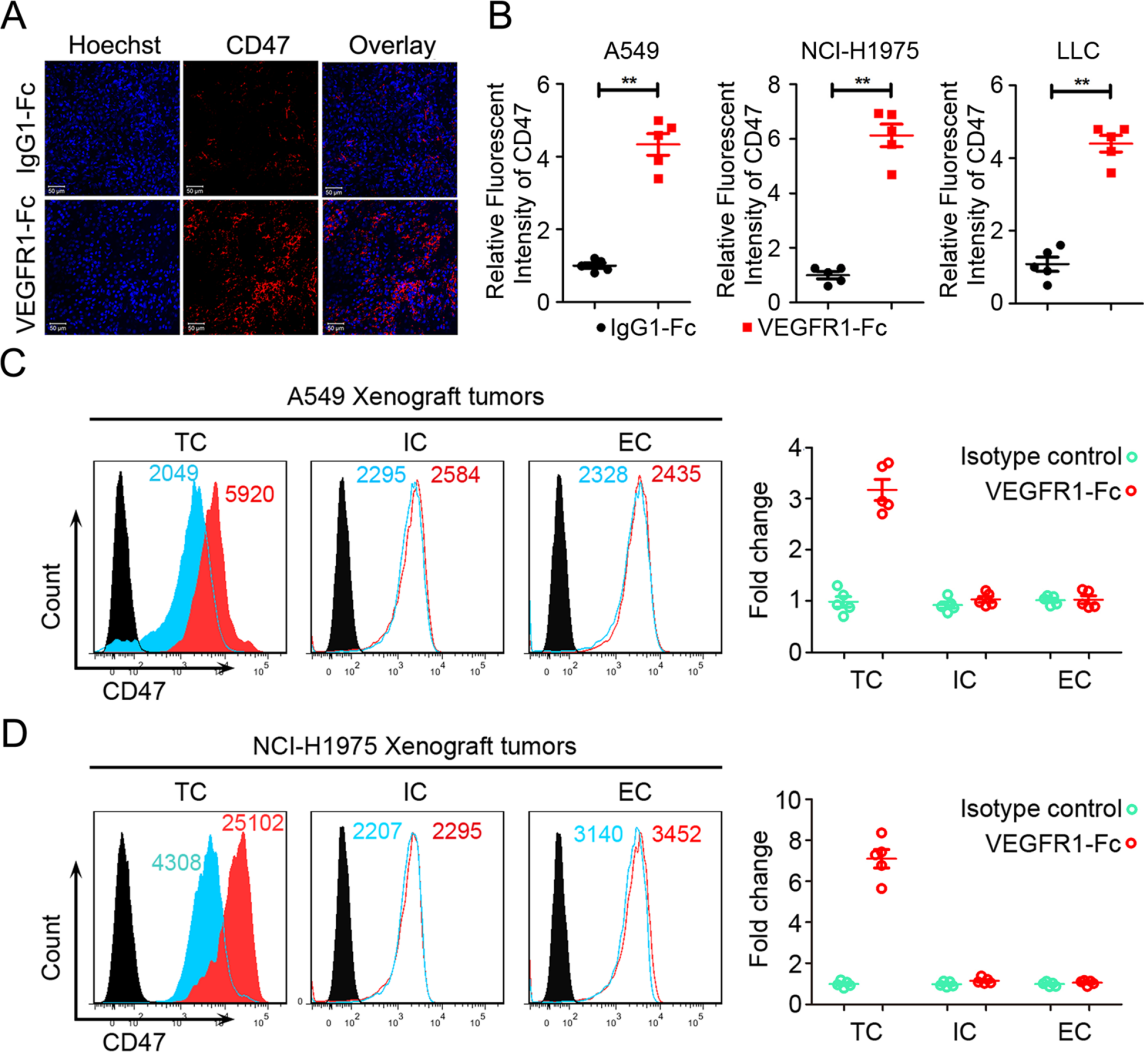
VEGF/VEGFR blockade increased CD47 expression on NSCLC cells. **a** and **b** PE-labelled anti-CD47 antibody was used to detect the CD47 expression in the tissues of A549 (**a**), NCI-H1975 and LLC tumors. **c** and **d** FACS analysis of CD47^+^ cell composition of A549 (**c**) and NCI-H1975 (**d**) tumor models treated with IgG1-Fc and VEGFR1-Fc. TC: tumor cell, IC: immune cell, EC: endothelial cell. (*N* = 5 per group, each point indicated a value from one mouse)

To determine innate immune-related underpinnings of resistance to anti-angiogenic treatment in NSCLC, we used A549, NCI-H1975 and LLC tumor models with responses to VEGF inhibitors. As shown in (Additional file 1: Figure S1), anti-angiogenic treatment (VEGFR1-Fc fusion protein, or anti-VEGF antibody bevacizumab) could tentatively control tumor growth for about 2 to 3 weeks followed by resistance to anti-angiogenic therapy and robust tumor growth, and finally did not elicit significant survival benefits (Additional file 2: Figure S2). Immunofluorescence staining of immune checkpoint regulator in NSCLC models revealed a significant increased expression of CD47 in refractory NSCLC (Fig. 1a and b, Additional file 3: Figure S3, and Additional file 4: Figure S4). Fluorescence-activated cell sorting (FACS) and immunoblot analysis showed that tumor cells were the primary source of CD47 increased cells in NSCLC (Fig. 1c and d, and Additional file 5: Figure S5). In brief, these data showed that CD47 was up-regulated by anti-angiogenic therapy in a tumor cell-specific manner.

### Anti-angiogenic therapy increased CD47 via TNF-α/NF-κB1

**Fig. 2:**
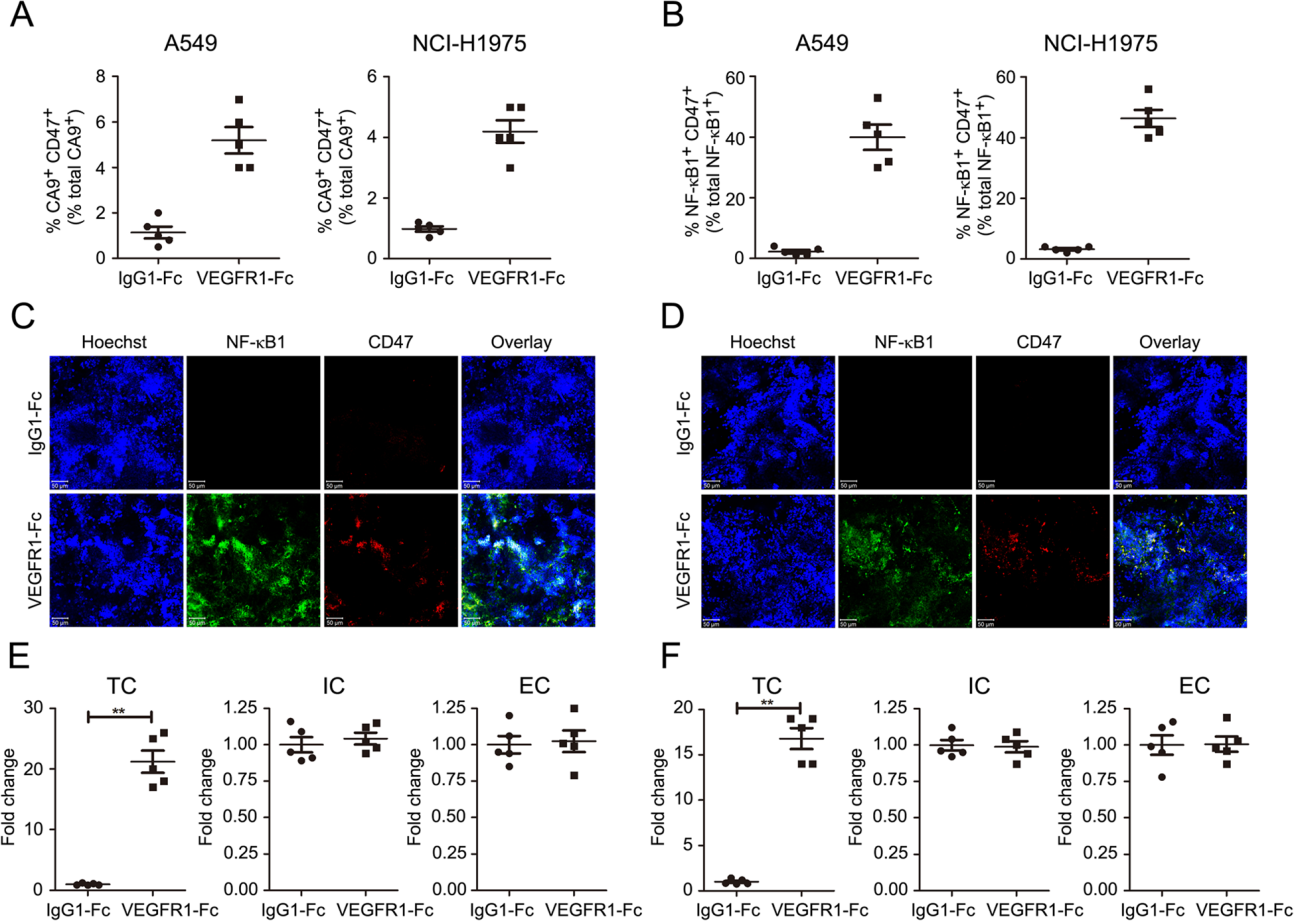
Anti-angiogenic treatment activated TNF-α/NF-κB1 pathway in NSCLC cells. **a** Quantitation of CA9 and CD47 in NSCLC xenograft tumors treated with IgG1-Fc or angiogenesis inhibitor. **b** Quantitation of NF-κB1 and CD47 in tumors treated with IgG1-Fc or angiogenesis inhibitors. **c** and **d** Immunofluorescence staining of NF-κB1 and CD47 in A549 (**c**) and NCI-H1975 (**d**) xenograft tumor tissues. **e** and **f** Quantitative polymerase chain reaction (qPCR) analysis of *TNF-α* in FACS-sorted TCs, ECs and ICs from A549 (**e**) and NCI-H1975 (**f**) xenograft tumors. (** *P* < 0.01, *N* = 5 per group, each point indicated an independent value)

**Fig. 3:**
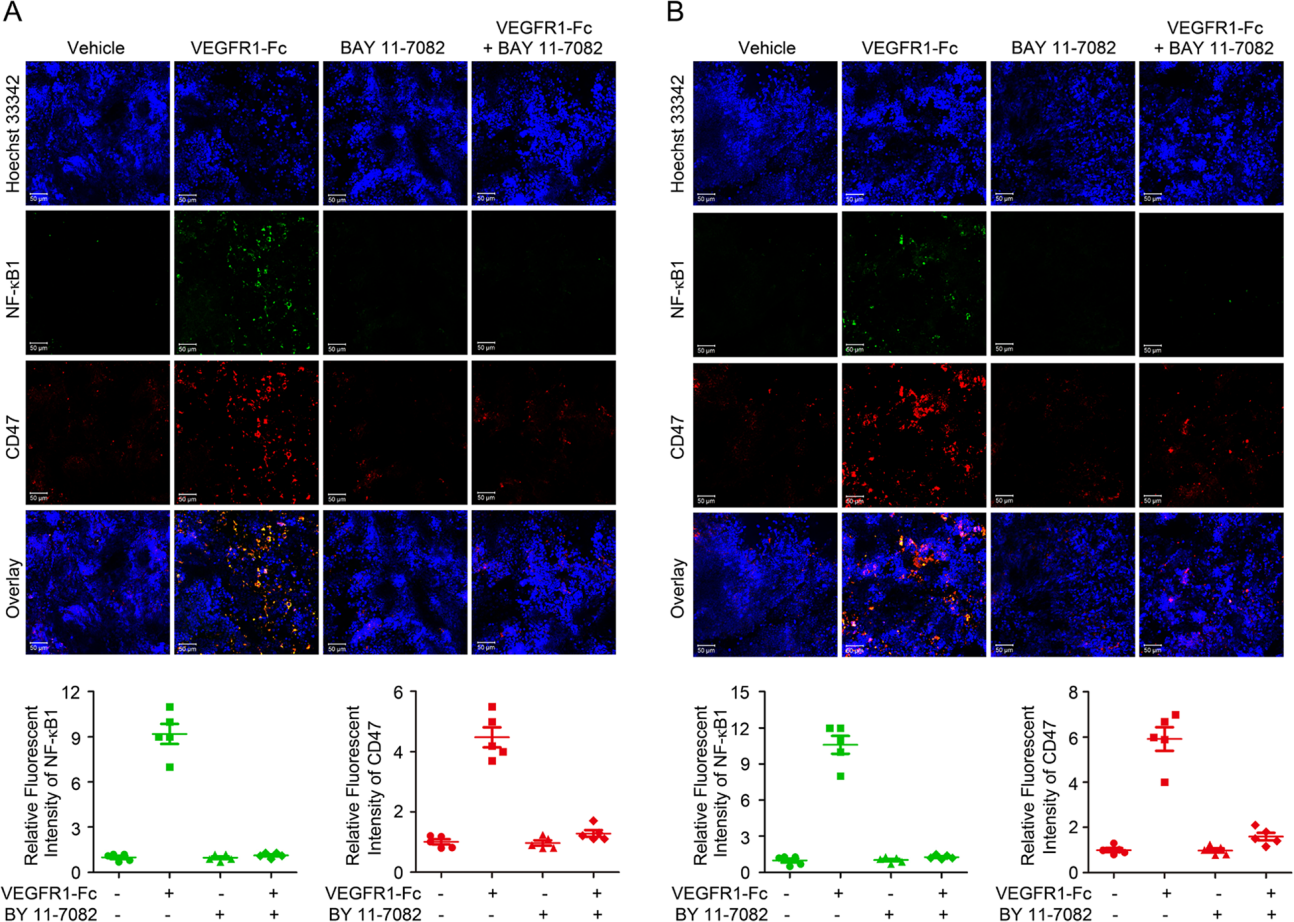
Blocking TNF-α/NF-κB1 reversed VEGFR1-Fc-induced CD47 upregulation. **a** and **b** Immunofluorescence staining and the relative fluorescent intensity of NF-κB1 and CD47 in A549 (**a**) and NCI-H1975 (**b**) xenograft tumor tissues (*N* = 5 per group, each point represented an independent value)

Then, we sought to investigate how VEGF inhibitor increased CD47 expression on NSCLC cells. Considering the fact that anti-angiogenic therapy could reduce vessel density and induce hypoxic areas and inflammation in tumors, we, at first, isolated tumor cells from NSCLC xenograft tumors in the mice treated with/without VEGFR1-Fc fusion protein continuously for 4 weeks. Staining with antibodies against the hypoxia-regulated CA9 (carbonic anhydrase IX) and CD47 were employed to evaluate the ratio of hypoxia NSCLC cells that displayed CD47 expression. Although VEGF inhibitor increased tumor hypoxia in A549 and NCI-H1975 xenograft models, there are only 4 to 6% of total CA9^+^ cells that were CD47^+^, demonstrating that hypoxia was not the major cause of CD47 upregulation during anti-angiogenic therapy (Fig. 2a). Because NF-κB1 transcription factor was one regulator that directly regulated CD47 expression, we examined the percentage of NF-κB1^+^ cells in this population and detected the co-localization of NF-κB1 and CD47. We found that 40 to 60% NF-κB1^+^ tumor cells were CD47^+^ (Fig. 2b-d). Then we isolated NF-κB1^+^ cells from these two xenograft models and sorted them into endothelial cells, immune cells and tumor cells, and researched the expression profile of the upstream of NF-κB1: TNF-α (tumor necrosis factor-alpha). As shown in Fig. 2e and f, VEGF blockade also substantially enhanced TNF-α expression in relapsing tumor cells. In addition, data from syngeneic immunocompetent tumor model also showed that VEGFR1-Fc treatment increased TNF-α/NF-κB1 pathway in refractory LLC tumors (Additional file 6: Figure S6). To evaluate the possible role of TNF-α/NF-κB1 pathway in anti-angiogenic therapy-induced CD47 upregulation, the tumor-bearing mice were simultaneously treated with VEGF inhibitor and BAY 11–7082 (TNF-α/NF-κB1 inhibitor). After TNF-α/NF-κB1 being successfully abrogated by BAY 11–7082, anti-angiogenic treatment-induced CD47 upregulation was diminished in NSCLC tumors and produced increased anti-tumor effect (Fig. 3a and b, Additional file 6: Figure S6).

These results demonstrated that TNF-α/NF-κB1 signaling pathway was involved in VEGF/VEGFR blockade-induced CD47 expression.

### CD47-SIRPα inhibition potentiated response to VEGF blockade in NSCLC

**Fig. 4:**
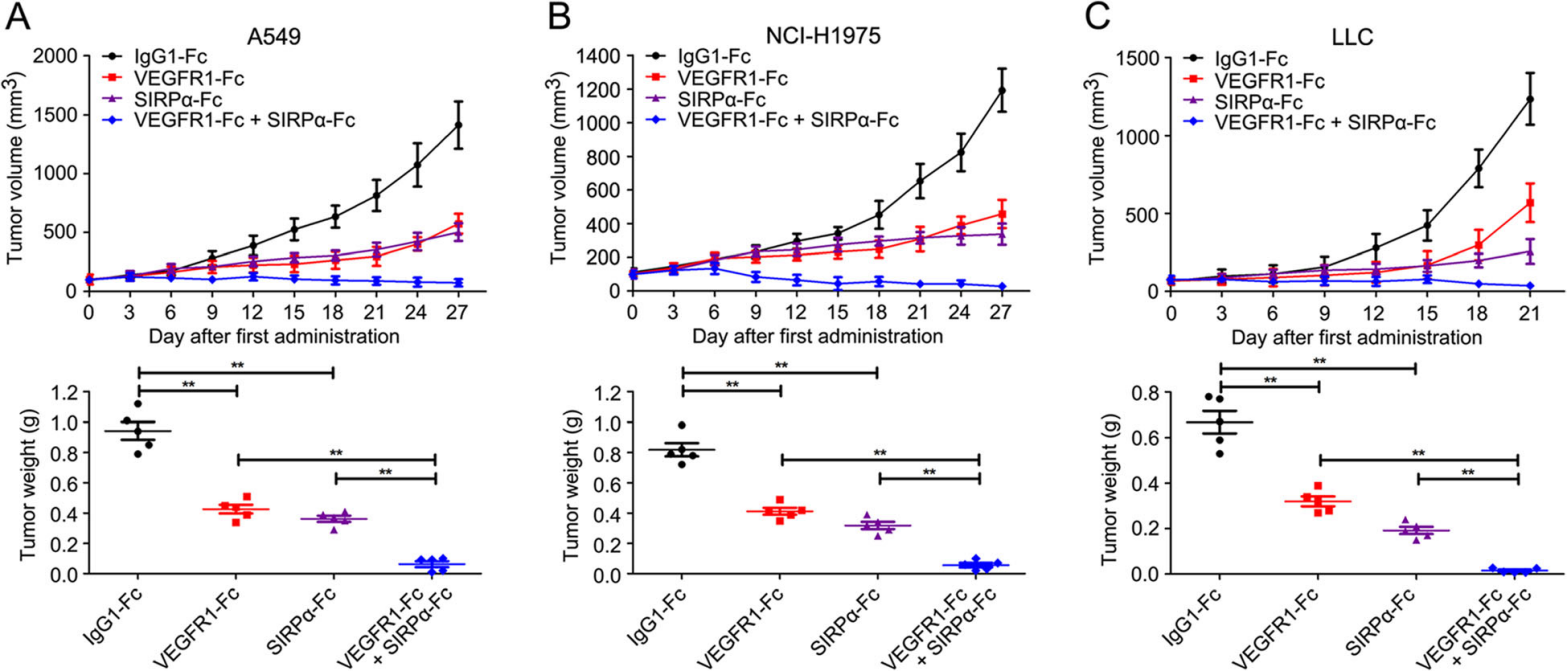
CD47 blocking therapy potentiated response to VEGF blockade in NSCLC. **a** and **b** In A549 (**a**) and NCI-H1975 (**b**) xenograft model, tumor volume was measured. After treatment with VEGFR1-Fc and/or SIRPα-Fc for 27 days, tumor weight was presented. **c** In LLC tumor model, tumor volume was presented. After treatment with VEGFR1-Fc and/or SIRPα-Fc, tumor weight was shown. (mean ± SD, *N* = 5 per group; ** *P* < 0.01)

Then, we speculated that inhibition of CD47 could be sufficient to extend an anti-tumor response during anti-angiogenic treatment. To examine this proposition, we treated NSCLC xenograft mice with VEGFR1-Fc alone or in combination with SIRPα-Fc. After a temporal remission, tumors became refractory as characterized by increased tumor burden after 2 to 3 weeks of VEGFR1-Fc treatment. In contrast, comparable to VEGFR1-Fc monotherapy, anti-angiogenic therapy in combination with CD47 blockade inhibited tumor regrowth and resulted in a low tumor burden (Fig. 4). In A549 xenograft model, tumor weight in VEGFR1-Fc group was 426.04 ± 64.26 mg versus 942.20 ± 130.27 mg of the isotype control (*P* < 0.0001) (Fig. 4a), and the tumor weight in VEGFR1-Fc and SIRPα-Fc co-treatment group was 68.15 ± 35.64 mg (*P* < 0.0001 versus VEGFR1-Fc group). In NCI-H1975 tumor model, tumor weight in mice co-treated with VEGFR1-Fc and SIRPα-Fc was 56.08 ± 32.09 mg (*P* < 0.0001 versus VEGFR1-Fc cohort), while the tumor weight in VEGFR1-Fc group and the control were 412.15 ± 51.19 mg and 818.09 ± 97.57 mg, respectively (Fig. 4b). In LLC tumor models, tumor weight in VEGFR1-Fc and SIRPα-Fc co-treatment group was 15.11 ± 9.03 mg versus 320.02 ± 43.3 mg of VEGFR1-Fc group (*P* < 0.0001) (Fig. 4c).

In brief, these results showed that blocking CD47 by SIRPα-Fc potentiated anti-tumor response of NSCLC to VEGF blockade.

### Targeting CD47 increased macrophage phagocytosis of NSCLC cells relapsing from anti-angiogenic therapy

**Fig. 5:**
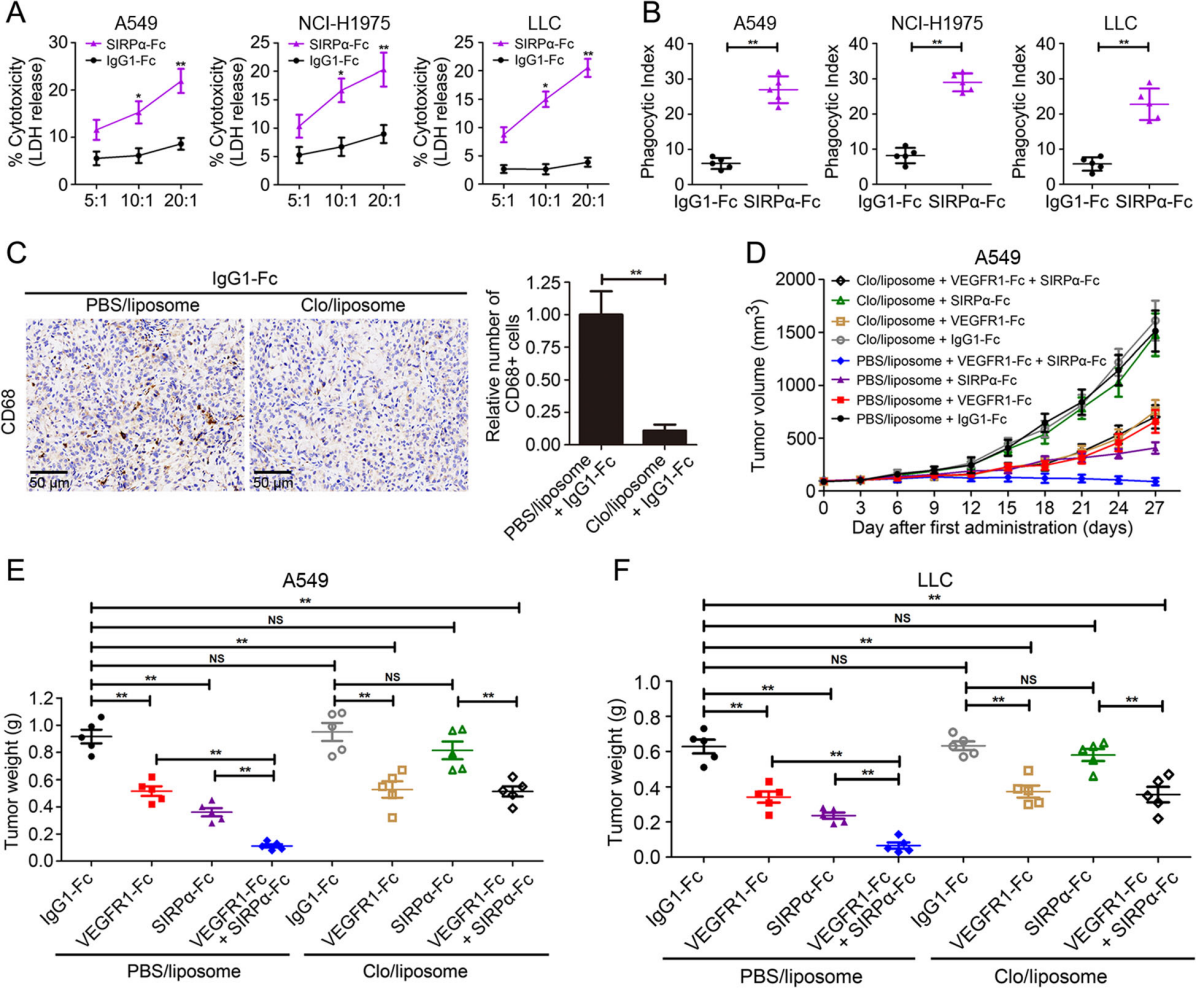
Targeting CD47 elicited macrophage cytotoxicity and phagocytosis against relapsed NSCLC cells. **a** SIRPα-Fc elicited macrophage cytotoxicity against relapsing A549, NCI-H1975 and LLC cells under various effector: target cell ratio. **b** SIRPα-Fc increased macrophage phagocytosis of relapsing A549, NCI-H1975 and LLC cells. (Each point repeated a value from one independent experiment and data were shown as mean ± SD). **c** A549 or LLC tumor models were established. CD68 staining was employed to detect macrophage depletion. **d** Tumor volume and tumor weight (**e** and **f**) were measured and shown as mean ± SD. (*N* = 5 per group). NS: no significance; * *P* < 0.05, ** *P* < 0.01

The NF-κB1 response in NSCLC undergoing VEGF/VEGFR blockade induced a negative feedback loop. The feedback loop increased CD47 expression that deactivated macrophage activity via binding to SIRPα, thereby rendering tumors more immunosuppressive. Then, we isolated tumor cells from NSCLC tumors in VEGFR1-Fc-treated mice and investigated whether targeting CD47 could eliminate the relapsing NSCLC cells. SIRPα-Fc fusion protein was used to disrupt CD47-SIRPα axis. SIRPα-Fc alone showed negligible effects on the cell viability (data not shown). While SIRPα-Fc could increase macrophage cytotoxicity against the relapsing NSCLC cells (Fig. 5a). Compared with isotype control IgG1-Fc, SIRPα-Fc increased the phagocytic index from 6.0 to 27.0, from 8.0 to 29.0 and from 6.0 to 23.0 in the A549 cells, NCI-H1975 cells and LLC cells relapsing from anti-angiogenic therapy, respectively (Fig. 5b). Furthermore, to detect the relevant of macrophages and CD47 in vivo, Clo/liposome (clodronate liposome) was employed to deplete macrophages in NSCLC xenograft model (Fig. 5c). Compared with the negative control PBS/liposome, Clo/liposome accelerated the tumor growth of mice treated with SIRPα-Fc (Fig. 5d, e and Additional file 7: Figure S7a). Tumor weight in PBS/liposome + IgG1-Fc group, PBS/liposome + VEGFR1-Fc group, PBS/liposome + SIRPα-Fc group, PBS/liposome + VEGFR1-Fc + SIRPα-Fc group were 916.62 ± 113.49 mg, 516.00 ± 78.29 mg, 360.20 ± 68.34 mg, 112.12 ± 28.84 mg. While tumor weight in Clo/liposome + IgG1-Fc group, Clo/liposome + VEGFR1-Fc group, Clo/liposome + SIRPα-Fc group and Clo/liposome + VEGFR1-Fc + SIRPα-Fc group were 950.01+ 147.82 mg, 528.16 + 134.24 mg, 814.66 ± 145.58 mg and 513.98 + 84.44 mg. These data showed that macrophage depletion totally abrogated the anti-tumor effect of SIRPα-Fc.

In addition, syngeneic immunocompetent tumor model was established to confirm the relevant of macrophage and CD47 was also evaluated in LLC tumors. We found that Clo/liposome recovered the tumor burden in mice treated with SIRPα-Fc. Tumor weight in Clo/liposome + SIRPα-Fc group was 580.02 + 76.82 mg versus 236.86 + 39.45 mg of PBS/liposome + SIRPα-Fc group (*P* < 0.001), and tumor weight in Clo/liposome + VEGFR1-Fc + SIRPα-Fc group was 355.78 + 98.91 mg versus 67.54 + 41.18 mg of PBS/liposome + VEGFR1-Fc + SIRPα-Fc group (*P* < 0.001). While the tumor weight in Clo/liposome + IgG1-Fc group and PBS/liposome + IgG1-Fc group were 632.16 + 55.96 mg and 628.38 + 86.98 mg, respectively (Fig. 5f and Additional file 7: Figure S7). These results uncovered that blocking CD47 by SIRPα-Fc could induce effective macrophage-mediated elimination of the relapsing NSCLC cells.

### Co-targeting CD47 and VEGF elicited synergetic anti-tumor effect in NSCLC and prolonged the median survival

**Fig. 6:**
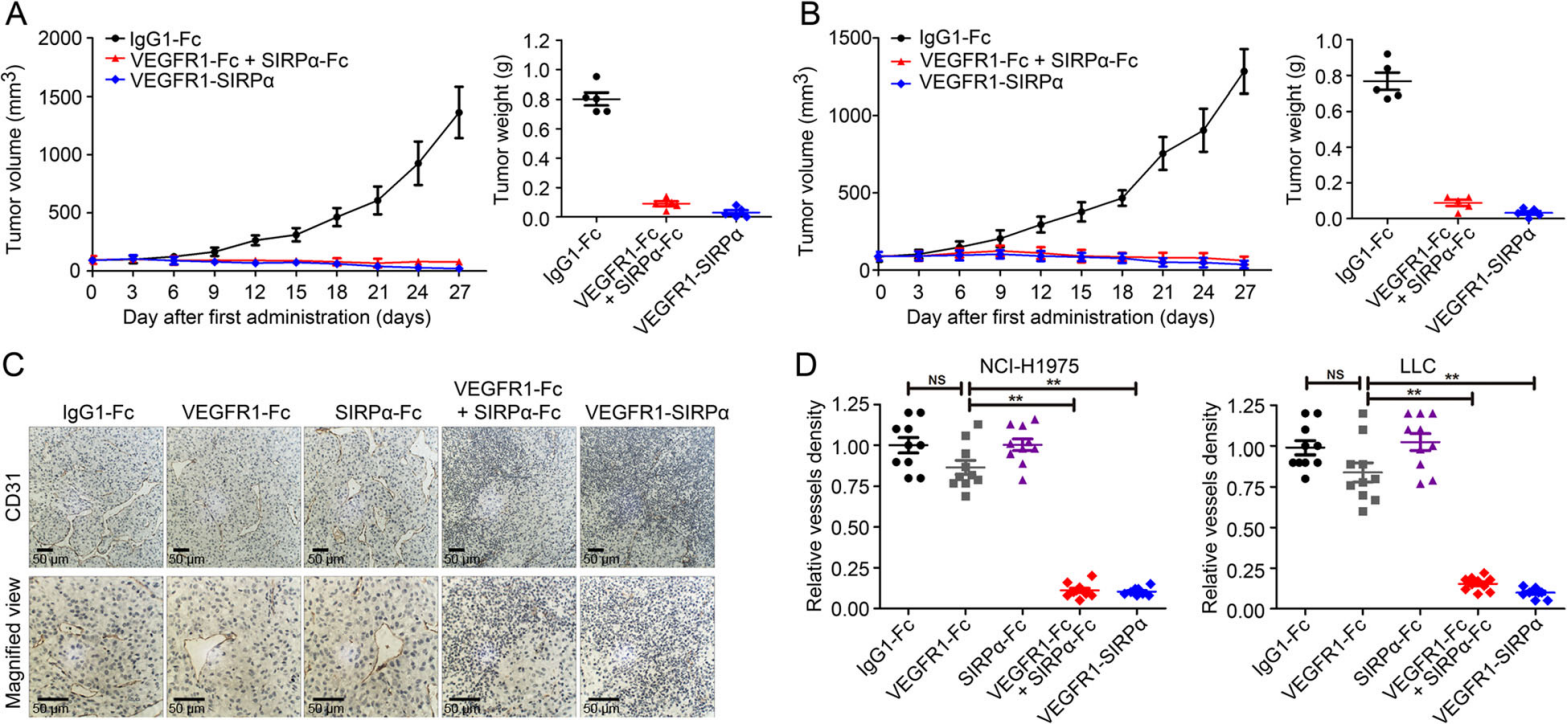
Co-targeting CD47 and VEGF elicited synergetic anti-tumor effects in NSCLC. **a** In A549 xenograft model, tumor volume was presented. After treatment with VEGFR1-SIRPα or VEGFR1-Fc plus SIRPα-Fc, tumor weight was shown (mean ± SD, *N* = 5 per group). **b** In NCI-H1975 xenograft model, tumor volume was measured. After treatment with VEGFR1-SIRPα or VEGFR1-Fc plus SIRPα-Fc, tumor weight was shown (mean ± SD, *N* = 5 per group). **c** Representative image of immunohistochemistry CD31 staining of NCI-H1975 xenograft tumor tissues. **d** The relative vessels density of NCI-H1975 tumor or LLC tumor tissues. NS: no significance; ** *P* < 0.01

**Fig. 7:**
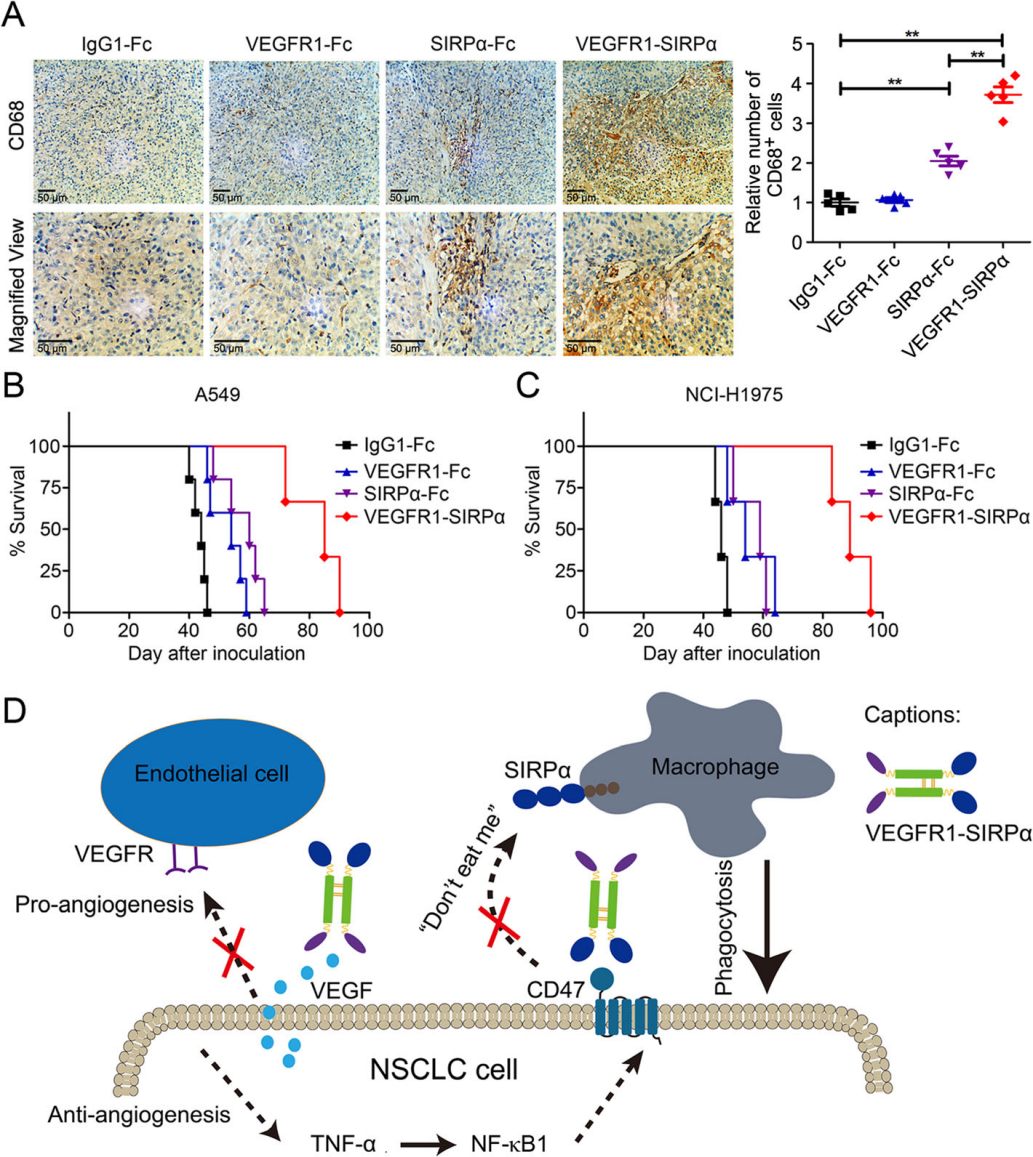
Targeting CD47 and VEGF significantly prolonged the median survival of NSCLC-bearing mice. **a** Immunohistochemistry CD68 staining of NCI-H1975 tumor tissues. **b** and **c** A549 metastatic model (**b**) and NCI-H1975 metastatic model (**c**) were constructed to challenge the effects of VEGFR1-SIRPα on the survival (*N* = 5 per group). **d** The description of combined anti-angiogenic and CD47-blocking therapies eliciting potent anti-tumor effect in NSCLC

Next, we aimed to evaluate the therapeutic effects of simultaneous disruption of angiogenetic axis and CD47/SIRPα axis in NSCLC. VEGFR1-SIRPα fusion protein was employed to target CD47 and VEGF simultaneously. In A549 xenograft model, tumor volume presented that targeting VEGF and CD47 by VEGFR1-SIRPα could elicited potent anti-tumor effect (Fig. 6a). After 27 days’ treatment, tumor weight in the groups of isotype control and VEGFR1-SIRPα were 802.05 ± 95.98 mg and 30.20 ± 34.64 mg, respectively. Similarly, in NCI-H1975 tumor model, tumor weight in the groups of isotype control and VEGFR1-SIRPα were 768.11 ± 107.56 mg and 32.00 ± 23.87 mg, respectively (Fig. 6b). Microvessels-specific marker CD31 was used to determine microvessel density and Fig. 6c and d presented that blocking CD47 potentiated the anti-angiogenic effects of VEGFR1-Fc (*P* < 0.01). In Fig. 7a, Additional file 8: Figure S8 and Additional file 9: Figure S9 histopathological analysis and flow cytometry plots demonstrated that VEGFR1-SIRPα elicited prominent macrophage infiltration without significant VEFGA production. Dendritic cells were also involved in CD47 blockade-induced anti-tumor effect in NSCLC (Additional file 9: Figure S9b). To assess whether blocking angiogenetic axis and CD47/SIRPα could extend the survival, two metastatic models were established. In A549 metastatic model, compared to the isotype control, VEGFR1-Fc showed no significant effect on the median survival. SIRPα-Fc group had a median survival of 60 days, whereas VEGFR1-SIRPα could extend the median survival to 85 days (Fig. 7b). In NCI-H1975 metastatic model, median survival of the mice treated with isotype control, VEGFR1-Fc, SIRPα-Fc, VEGFR1-SIRPα were 46 days, 54 days, 59 days and 89 days, respectively (Fig. 7c).

These data showed that blocking angiogenetic axis and CD47/SIRPα axis elicited synergetic anti-tumor effect in NSCLC and significantly prolonged the median survival via anti-angiogenesis and macrophage activation.

## Discussion

Although anti-angiogenic therapy could improve progression-free survival (PFS) in some NSCLC patients, but overall survival (OS) is modestly improved and most patients are unfortunately short-lived [6, 29–32]. Here, we revealed the up-regulation of CD47, the negative checkpoint molecule that binds to SIRPα, as an innate immunosuppressive mechanism that limited the anti-tumor effect of VEGF/VEGFR inhibitors. During the anti-angiogenic therapy, a negative feedback was created by upregulating CD47 to inactivate macrophage phagocytosis. Simultaneous blockade of angiogenetic axis and CD47/SIRPα axis significantly improved anti-tumor efficacy and prolonged median survival in NSCLC-bearing mice, which was most likely mediated through facilitating enhanced macrophage infiltration and producing successful extermination of experimental NSCLC.

Previous studies have reported that reinstating tumor growth by neovascularization or by modifying the growth behavior could help malignancy adapt to the limit of vascular growth restriction [10]. It has been demonstrated that these adaptations could also be regulated by the host immune system, which provided additional cytokines and chemokines that promoted angiogenesis and immunosuppression [33–35]. The expression of VEGF-A and PD-1/PD-L1 in lymph nodes of 103 patients were quantified and the data showed the higher positivity of VEGF-A and PD-1 in metastatic nodes and the surrounding negative nodes in comparison with non-metastatic patients [36]. Notably, in pancreatic (RT2-PNET, pancreatic neuroendocrine tumors), breast (PyMT, polyoma middle T oncoprotein) and brain (GBM, glioblastoma) tumor mouse models, expression of PD-L1, the ligand of PD-1, was enhanced by interferon-γ-expressing T cells in tumors relapsed from VEGF-A inhibition [9]. The above studies mainly focused on investigating the adaptive immune system in anti-angiogenic therapy. In the current work, we studied the important role of innate immune response in anti-angiogenic therapy and described for the first time that the upregulation of CD47 as a result of anti-angiogenic therapy plays an important role in relapsing NSCLC.

Our study showed that anti-angiogenic treatment-induced negative feedback, facilitating the interaction of CD47^+^ NSCLC cells with innate immune cells, was in line with the previous observation that the anti-tumor effects of anti-angiogenic therapy depended on the immunostimulatory environment formation [16]. As a key anti-phagocytic axis, CD47-SIRPα connection transfers “don’t eat me” signal to macrophage and inactivates macrophage phagocytosis, rendering cancer cell resistant to host’s innate immune monitoring [37]. Disrupting CD47/SIRPα signaling transduction by blocking antibodies (Hu5F9-G4 and CC-90002) could increase macrophage phagocytosis of multiple tumor cells and has been proved as a promising immunotherapeutic method for melanoma, breast cancer, small cell lung cancer and acute myeloid leukemia [38, 39]. Late-breaking studies reported that targeting CD47 by SIRPα-based fusion protein increased macrophage-mediated elimination of NSCLC and glioblastoma cells [25, 26]. Consistent with these studies, SIRPα-Fc was used to block the increased CD47 and was shown to trigger macrophage phagocytosis and cytotoxicity against NSCLC cells relapsing from anti-angiogenic treatment. Mechanistically, the combination of anti-angiogenic treatment and CD47 blockade could counteract the anti-angiogenic treatment-induced immunosuppressive pathway (CD47 up-regulation), and it was conceivable that CD47 blockade recruited and activated macrophages during anti-angiogenic therapy, eliciting enhanced anti-tumor efficacy.

Furthermore, coinstantaneous blocking VEGF and CD47 by VEGFR1-SIRPα fusion protein induced macrophages infiltration and CD47 blockade sensitized tumors to anti-angiogenic therapy. However, one important question that has yet to be answered is: what was the mechanism by which CD47 became upregulated on NSCLC cells relapsing from anti-angiogenic therapy. To answer this question, we isolated tumor cells from NSCLC tumors in VEGFR1-Fc-treated mice to elucidate the underlying mechanism. For the first time, we revealed that VEGF/VEGFR blockade-increased CD47 expression was dependent on the activation of TNF-α/NF-κB1 signaling pathway. Our results were consistent with previous study indicating that CD47 was regulated by sets of pro-inflammatory super-enhancers in breast cancer, diffuse large B-cell lymphoma and acute lymphoblastic leukemia [40].

## Conclusions

This study demonstrated that the up-regulation of an innate immunosuppressive pathway was served as a resistant mechanism during anti-angiogenic therapy, by which CD47 was enhanced via TNF-α/NF-κB1 signal pathway in refractory lung tumor models following anti-angiogenic therapy. Simultaneously disrupting CD47/SIRPα anti-phagocytic axis and VEGF/VEGFR angiogenetic axis elicited macrophages infiltration and sensitized tumors to anti-angiogenic therapy (Fig. 7d). These results provided a novel insight into the resistant mechanisms in anti-angiogenic therapy, facilitating clinic application of VEGF/VEGFR inhibitors in combination with CD47-targeting immune checkpoint inhibitors.

## Supplementary information

**Additional file 1: Figure S1.** The anti-tumor effects of VEGF/VEGFR inhibitors in NSCLC.

**Additional file 2: Figure S2.** NSCLC metastasis models were constructed to assess the effect of VEGFR1-Fc on the survival.

**Additional file 3: Figure S3.** The expression of CD47 on NSCLC cells was detected by flow cytometry.

**Additional file 4: Figure S4.** CD47 expression was up-regulated by VEGF inhibitor in A549, NCI-H1975 and LLC tumors.

**Additional file 5: Figure S5.** The CD47 expression was up-regulated by VEGF inhibitor in different cell constituents in a tumor type-specific manner.

**Additional file 6: Figure S6** Targeting TNF-α/NF-κB1 reversed VEGFR1-Fc-induced CD47 upregulation in LLC tumors.

**Additional file 7: Figure S7.** SIRPα-Fc induced potent macrophage-mediated elimination of NSCLC cells.

**Additional file 8: Figure S8.** Flow cytometry profile of macrophages in NSCLC tumors treated with VEGFR1-Fc and/or SIPRα-Fc.

**Additional file 9: Figure S9.** (a) FACS analysis was employed to sort CD68^+^ macrophages from LLC tumor and the VEGFA level in CD68^+^ macrophage was measured. SIRPα-Fc enhanced macrophage infiltration without significant VEFGA production in the tumors. (b) CD11c was used as a marker to detect dendritic cells in LLC tumor treated with SIRPα-Fc or VEGFR1-SIRPα.

## Data Availability

All data generated and analyzed during this study are included in this published article and its supplementary information.

## Abbreviations

CD47: Cluster of differentiation 47
CFDA SE: Carboxyfluorescein diacetate succinimidyl ester
GBM: Glioblastoma
LLC: Lewis Lung Carcinoma
NSCLC: Non-small cell lung cancer
OS: Overall survival
PFS: Progression-free survival
SIRPα: Signal regulatory protein alpha
VEGFR: Vascular endothelial growth factor receptor
