# Supplementary material for: Blocking CD47 efficiently potentiated therapeutic effects of anti-angiogenic therapy in non-small cell lung cancer
Source: J Immunother Cancer. 2019 Dec 11;7:346. doi: 10.1186/s40425-019-0812-9 (PMC6907216; doi:10.1186/s40425-019-0812-9)
Supplement: Supplementary file 1 — Additional file 1: Figure S1. The anti-tumor effects of VEGF/VEGFR inhibitors in NSCLC. [file 40425_2019_812_MOESM1_ESM.docx]

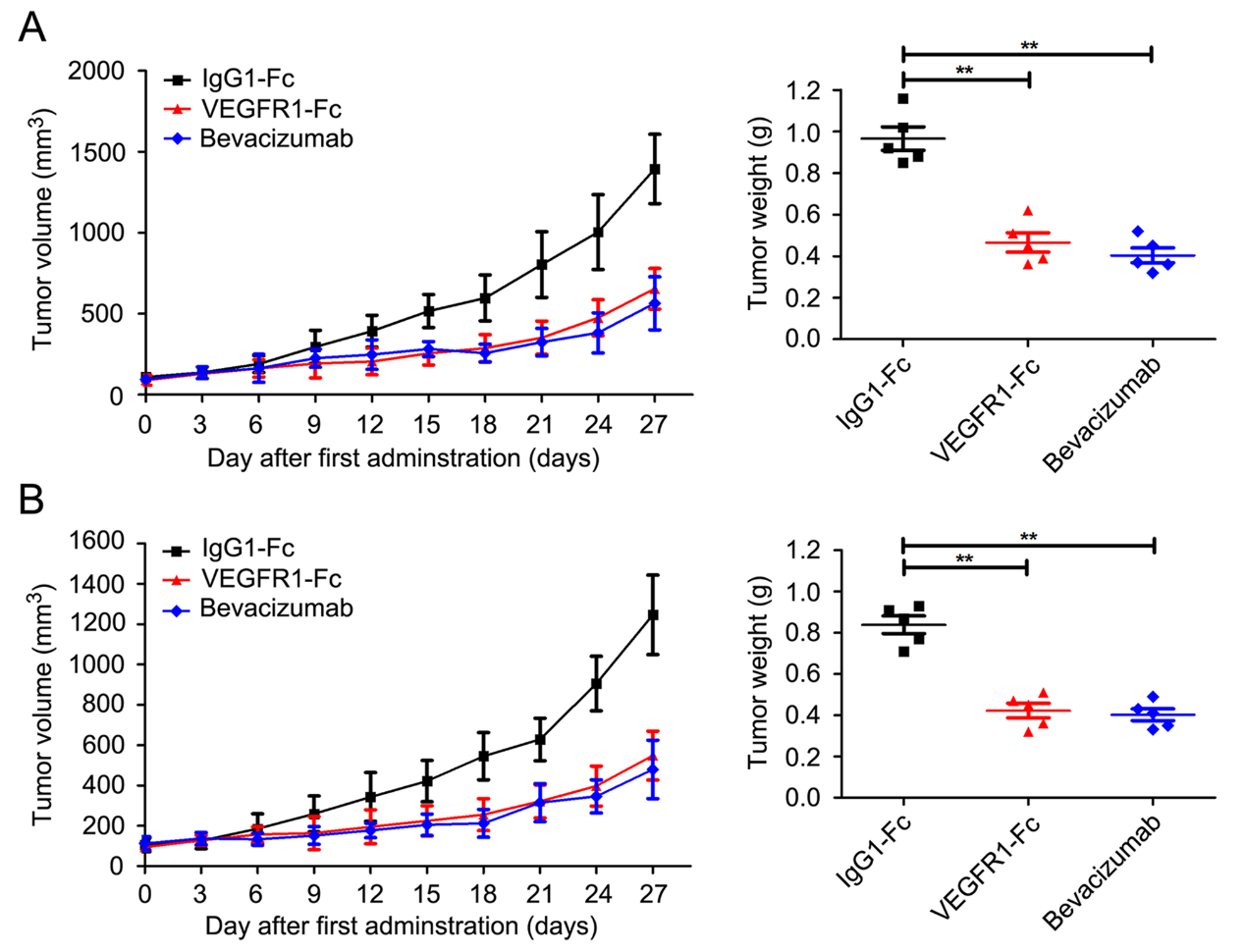


Supplementary Figure S1. The anti-tumor effects of VEGF/VEGFR inhibitors in NSCLC (a) A549 xenograft model, (b) NCI-H1975 xenograft model (*N* = 5 per group). Tumor weight was presented as mean ± SD and each point represented a value from an independent mouse. ** *P* < 0.01.
