# Supplementary material for: Blocking CD47 efficiently potentiated therapeutic effects of anti-angiogenic therapy in non-small cell lung cancer
Source: J Immunother Cancer. 2019 Dec 11;7:346. doi: 10.1186/s40425-019-0812-9 (PMC6907216; doi:10.1186/s40425-019-0812-9)
Supplement: Supplementary file 2 — Additional file 2: Figure S2. NSCLC metastasis models were constructed to assess the effect of VEGFR1-Fc on the survival. [file 40425_2019_812_MOESM2_ESM.docx]

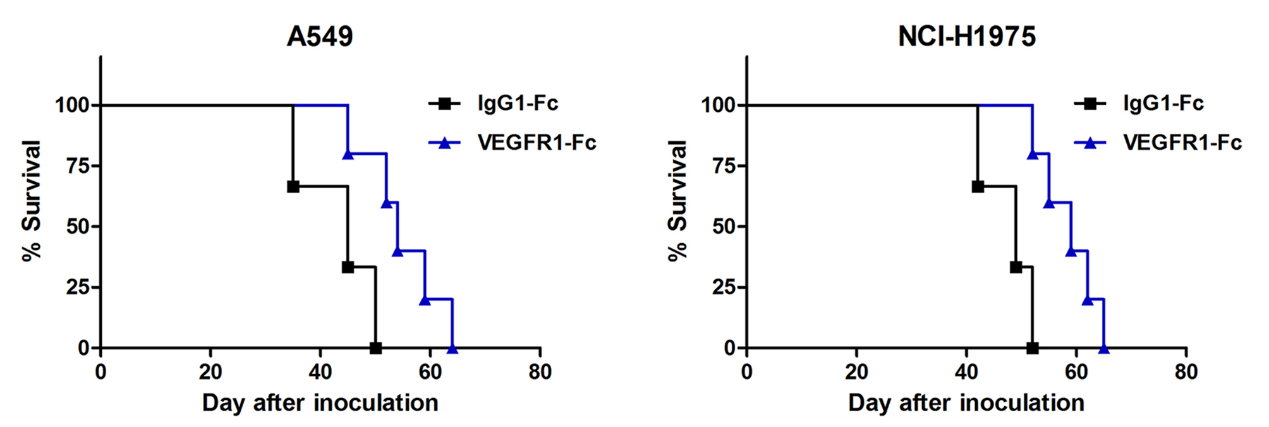


Supplementary Figure S2. NSCLC metastasis models were constructed to assess the effect of VEGFR1-Fc on the survival. Nude mice were injected with NSCLC cells (1 × 10^6^) via the tail vein to establish metastatic xenograft models. VEGFR1-Fc (10 mg/kg) and isotype control IgG1-Fc (10 mg/kg) were injected intraperitoneally twice a week (*N* = 5 per group).
