# Supplementary figures and images for: Blocking CD47 efficiently potentiated therapeutic effects of anti-angiogenic therapy in non-small cell lung cancer
Source: J Immunother Cancer. 2019 Dec 11;7:346. doi: 10.1186/s40425-019-0812-9 (PMC6907216; doi:10.1186/s40425-019-0812-9)

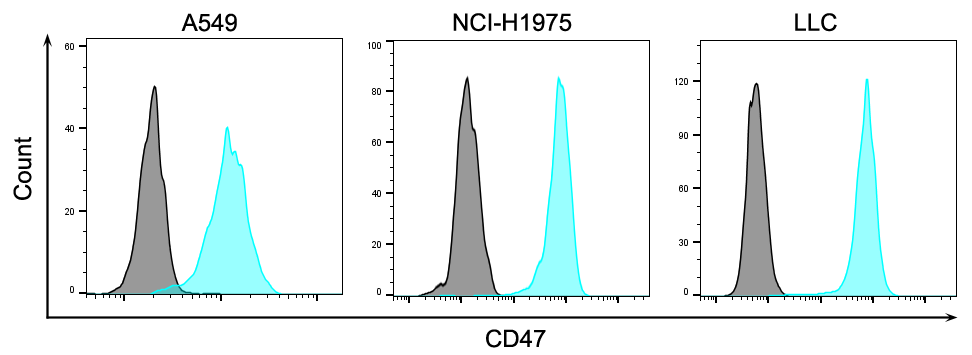


Supplementary Figure S3. The expression of CD47 on NSCLC cells was detected by flow cytometry.

Supplement: Supplementary file 3 — Additional file 3: Figure S3. The expression of CD47 on NSCLC cells was detected by flow cytometry. [file 40425_2019_812_MOESM3_ESM.docx]
