# Supplementary material for: Blocking CD47 efficiently potentiated therapeutic effects of anti-angiogenic therapy in non-small cell lung cancer
Source: J Immunother Cancer. 2019 Dec 11;7:346. doi: 10.1186/s40425-019-0812-9 (PMC6907216; doi:10.1186/s40425-019-0812-9)
Supplement: Supplementary file 4 — Additional file 4: Figure S4. CD47 expression was up-regulated by VEGF inhibitor in A549, NCI-H1975 and LLC tumors. [file 40425_2019_812_MOESM4_ESM.docx]

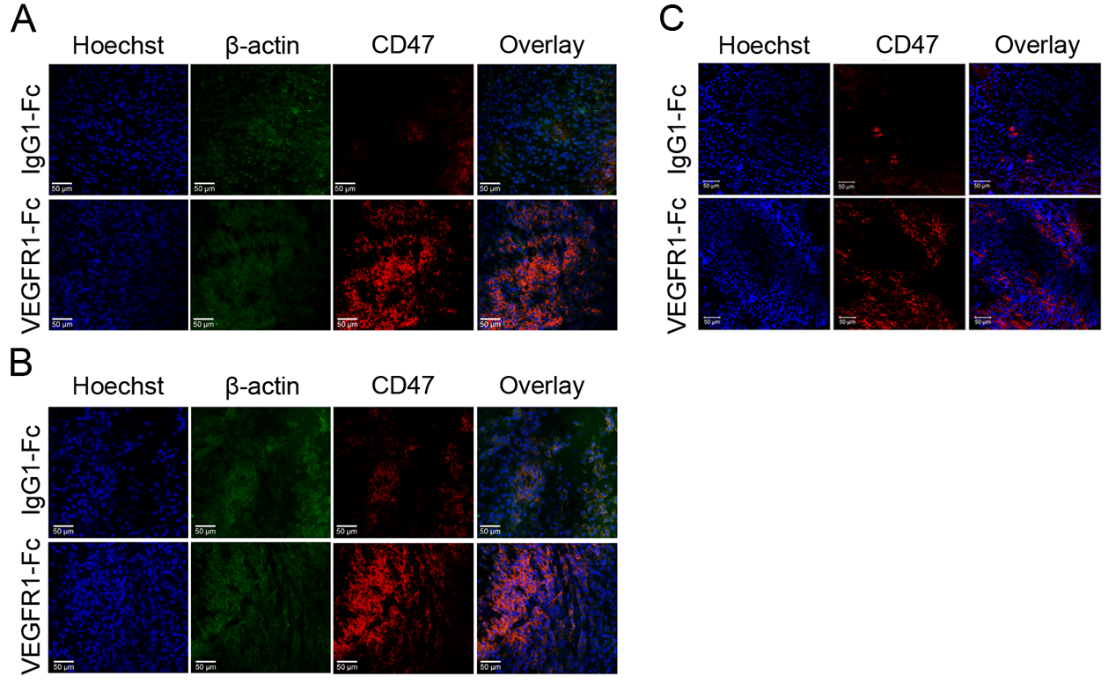


Supplementary Figure S4. CD47 expression was up-regulated by VEGF inhibitor in A549, NCI-H1975 and LLC tumors. Hoechst and Alexa Fluor 488-labeled anti-β-actin antibody was employed to locate the cells and PE-labelled anti-CD47 antibody was used to detect the CD47 expression in the tissues of A549 (a), NCI-H1975 (b) and LLC (c) tumors (*N* = 5 per group and each point indicated an independent value).
