# Supplementary material for: Blocking CD47 efficiently potentiated therapeutic effects of anti-angiogenic therapy in non-small cell lung cancer
Source: J Immunother Cancer. 2019 Dec 11;7:346. doi: 10.1186/s40425-019-0812-9 (PMC6907216; doi:10.1186/s40425-019-0812-9)
Supplement: Supplementary file 5 — Additional file 5: Figure S5. The CD47 expression was up-regulated by VEGF inhibitor in different cell constituents in a tumor type-specific manner. [file 40425_2019_812_MOESM5_ESM.docx]

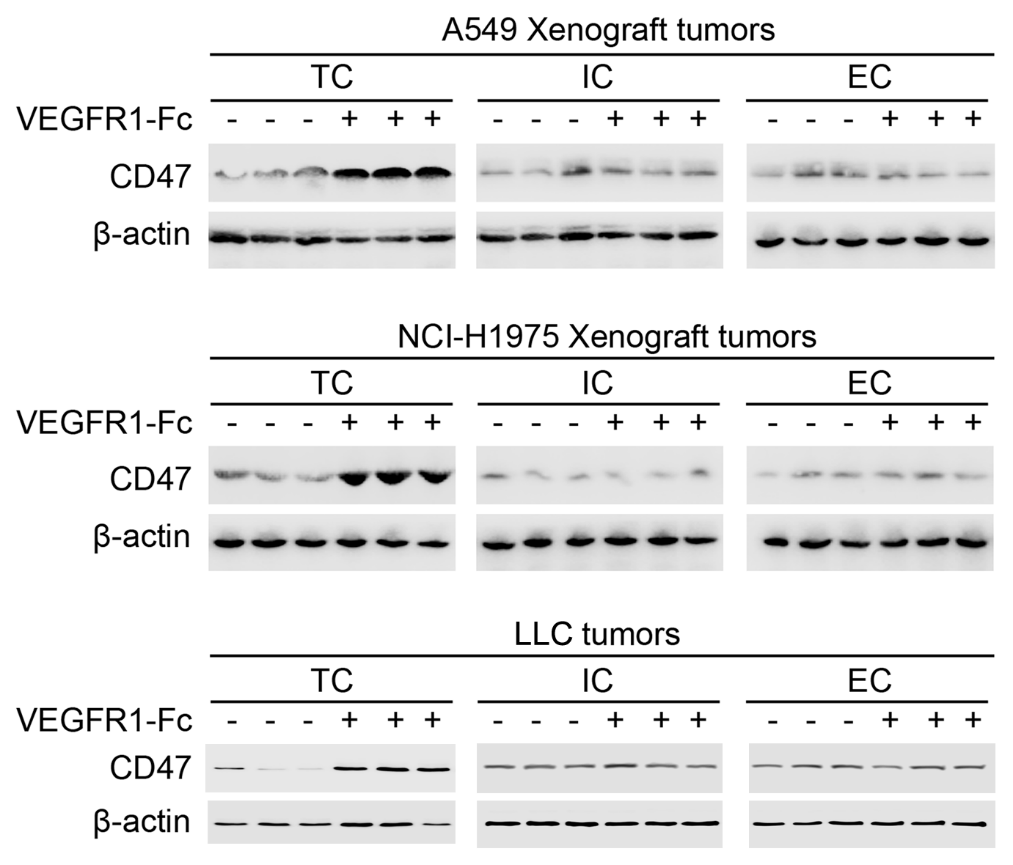


Supplementary Figure S5. The CD47 expression was up-regulated by VEGF inhibitor in different cell constituents in a tumor type-specific manner. (TC: tumor cells, IC: immune cells, EC: endothelial cells)
