# Supplementary material for: Blocking CD47 efficiently potentiated therapeutic effects of anti-angiogenic therapy in non-small cell lung cancer
Source: J Immunother Cancer. 2019 Dec 11;7:346. doi: 10.1186/s40425-019-0812-9 (PMC6907216; doi:10.1186/s40425-019-0812-9)
Supplement: Supplementary file 6 — Additional file 6: Figure S6 Targeting TNF-α/NF-κB1 reversed VEGFR1-Fc-induced CD47 upregulation in LLC tumors. [file 40425_2019_812_MOESM6_ESM.docx]

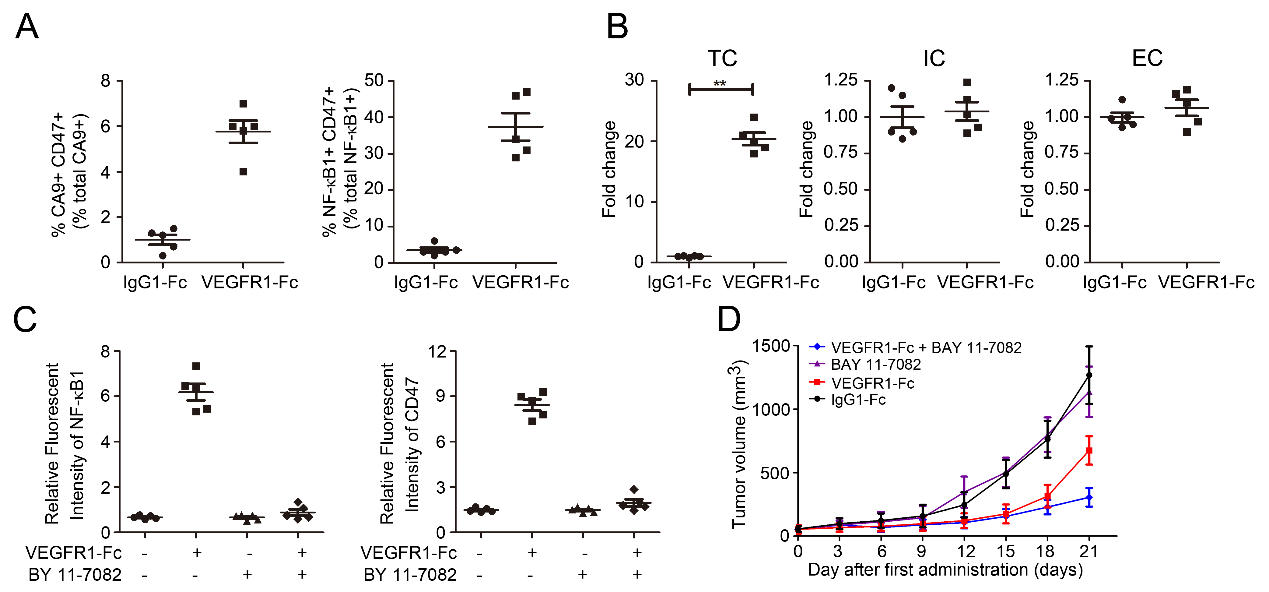


Supplementary Figure S6. Targeting TNF-α/NF-κB1 reversed VEGFR1-Fc-induced CD47 upregulation in LLC tumors. (a) Quantitation of CA9^+^CD47^+^ cells and NF-κB1^+^CD47^+^ cells in LLC tumors treated with IgG1-Fc or angiogenesis inhibitor. (b) qPCR analysis of *TNF-α* in FACS-sorted TCs, ECs and ICs from LLC tumors. ** *P* < 0.01. (c) Analysis of NF-κB1 and CD47 in LLC tumor tissues. (d) TNF-α/NF-κB1 inhibitor enhanced the anti-tumor effect of VEGFR1-Fc. (*N* = 5 per group and each point represented a value from one mouse).
