# Supplementary material for: Blocking CD47 efficiently potentiated therapeutic effects of anti-angiogenic therapy in non-small cell lung cancer
Source: J Immunother Cancer. 2019 Dec 11;7:346. doi: 10.1186/s40425-019-0812-9 (PMC6907216; doi:10.1186/s40425-019-0812-9)
Supplement: Supplementary file 7 — Additional file 7: Figure S7. SIRPα-Fc induced potent macrophage-mediated elimination of NSCLC cells. [file 40425_2019_812_MOESM7_ESM.docx]

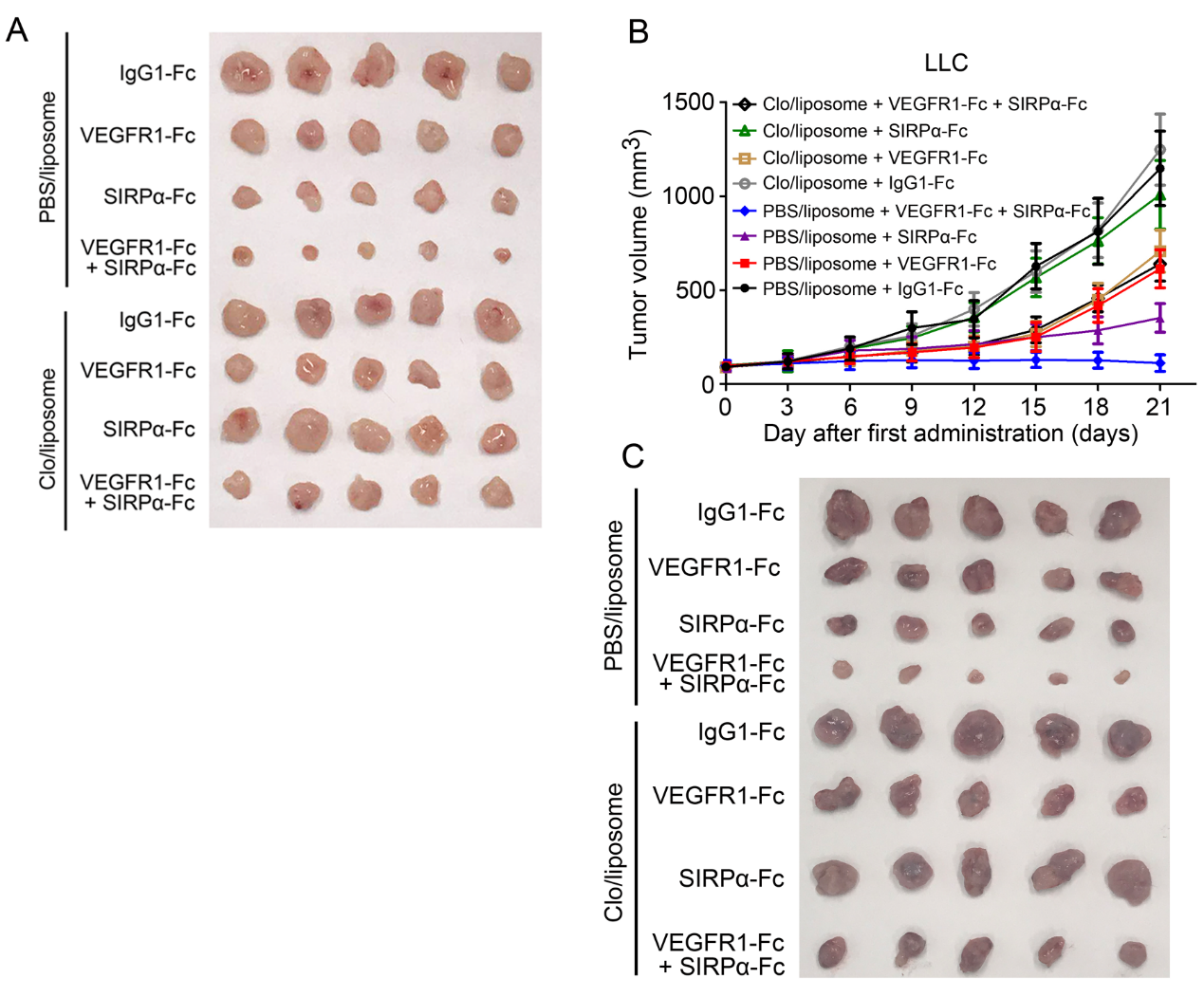


Supplementary Figure S7. SIRPα-Fc induced potent macrophage-mediated elimination of NSCLC cells. (a) BALB/c nude mice were injected subcutaneously with A549 cells to establish NSCLC xenograft model. Seven days later, tumor-bearing mice were randomly assigned into the indicated groups. Clo/liposome (200 μl per mouse) was injected intraperitoneally to deplete macrophages and PBS/liposome was used as a negative control. After treatment with SIRPα-Fc and/or VEGFR1-Fc for 27 days, the mice were sacrificed and tumors were collected. (b) C57BL/6 mice and LLC cells were employed to construct the immunocompetent model. Three days later, random allocation was taken to divide tumor-bearing mice into the indicated groups. Tumor volume was measured and the data were shown as mean ± SD. (c) After treatment with murine SIRPα-Fc and/or murine VEGFR1-Fc for 21 days, tumors were collected.
