# Supplementary material for: Blocking CD47 efficiently potentiated therapeutic effects of anti-angiogenic therapy in non-small cell lung cancer
Source: J Immunother Cancer. 2019 Dec 11;7:346. doi: 10.1186/s40425-019-0812-9 (PMC6907216; doi:10.1186/s40425-019-0812-9)
Supplement: Supplementary file 8 — Additional file 8: Figure S8. Flow cytometry profile of macrophages in NSCLC tumors treated with VEGFR1-Fc and/or SIPRα-Fc. [file 40425_2019_812_MOESM8_ESM.docx]

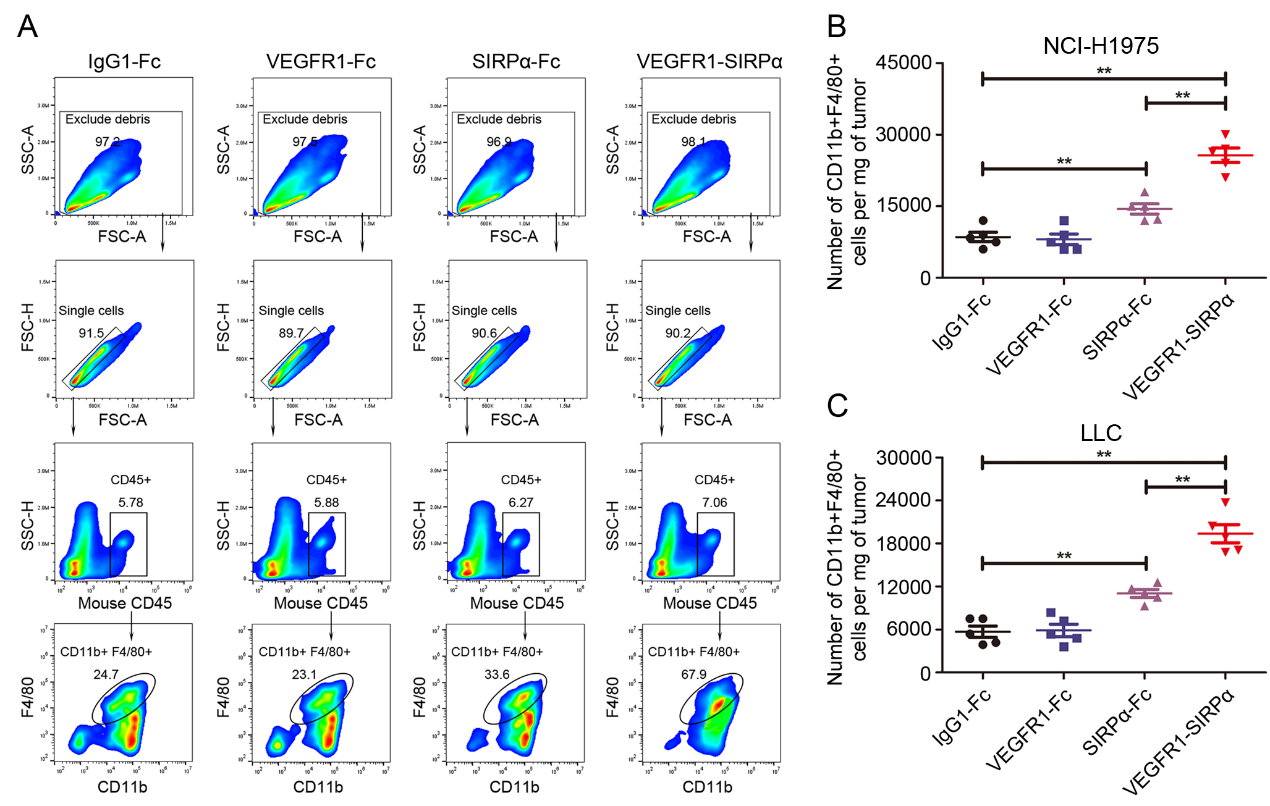


Supplementary Figure S8. Flow cytometry plots of macrophages in NSCLC tumors treated with VEGFR1-Fc and/or SIPRα-Fc. (a) Gate strategy and macrophages were defined as single, CD45^+^F4/80^+^CD11b^+^ cells. (b) The number of macrophages per mg of NCI-H1975 tumors treated with VEGFR1-Fc and/or SIPRα-Fc. (c) The number of macrophages per mg of LLC tumors treated with VEGFR1-Fc and/or SIPRα-Fc. (*N* = 5 per group, each point indicated a value from one mouse).
