# Supplementary material for: Blocking CD47 efficiently potentiated therapeutic effects of anti-angiogenic therapy in non-small cell lung cancer
Source: J Immunother Cancer. 2019 Dec 11;7:346. doi: 10.1186/s40425-019-0812-9 (PMC6907216; doi:10.1186/s40425-019-0812-9)
Supplement: Supplementary file 9 — Additional file 9: Figure S9. (a) FACS analysis was employed to sort CD68+ macrophages from LLC tumor and the VEGFA level in CD68+ macrophage was measured. SIRPα-Fc enhanced macrophage infiltration without significant VEFGA production in the tumors. (b) CD11c was used as a marker to detect dendritic cells in LLC tumor treated with SIRPα-Fc or VEGFR1-SIRPα. [file 40425_2019_812_MOESM9_ESM.docx]

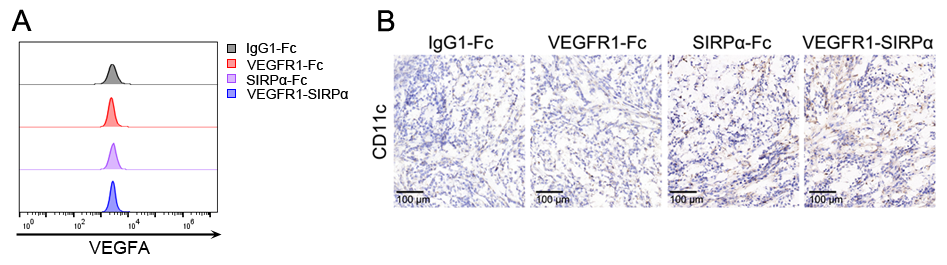


Supplementary Figure S9. (a) FACS analysis was employed to sort CD68^+^ macrophages from LLC tumor and the VEGFA level in CD68^+^ macrophage was measured. SIRPα-Fc enhanced macrophage infiltration without significant VEFGA production in the tumors. (b) CD11c was used as a marker to detect dendritic cells in LLC tumor treated with SIRPα-Fc or VEGFR1-SIRPα.
